# Efficacy of Oral Dydrogesterone in Improving Neonatal Birth Weight Outcomes in Idiopathic Intrauterine Growth Restriction: A Prospective Observational Study

**DOI:** 10.7759/cureus.90552

**Published:** 2025-08-20

**Authors:** Nadira Kasi, Naseeb Nama

**Affiliations:** 1 Obstetrics and Gynaecology, Sandeman Provisional Hospital, Quetta, Quetta, PAK; 2 Obstetrics and Gynaecology, Yeovil Hospital Somerset NHS Foundation Trust, Yeovil, GBR; 3 Obstetrics and Gynaecology, University Hospital of North Tees, Stockton-on-Tees, GBR

**Keywords:** birth weight, dydrogesterone, fetal growth retardation, pregnancy complications, progesterone, prospective studies, treatment outcome

## Abstract

Background and objective

Intrauterine growth restriction (IUGR) represents a significant obstetric complication with substantial global prevalence, with idiopathic cases constituting a considerable proportion where conventional therapeutic interventions demonstrate limited efficacy. Progesterone analogues have emerged as potential therapeutic agents for managing pregnancy-related complications, though evidence regarding their specific application in idiopathic IUGR remains limited. This prospective observational study aimed to evaluate the efficacy of oral dydrogesterone supplementation in improving neonatal birth weight outcomes among pregnant women diagnosed with idiopathic IUGR compared to conventional treatment modalities.

Methodology

A prospective observational study was conducted at the Department of Obstetrics and Gynecology, Sandeman Provincial Hospital, Quetta, over a six-month period from October 16, 2015, to April 15, 2016. The study enrolled 46 pregnant women aged 18-35 years with singleton pregnancies presenting at 28-34 weeks of gestation and diagnosed with idiopathic IUGR. Participants were allocated into two groups using a lottery method: Group A received conventional treatment comprising iron, folic acid supplementation, complete bed rest, and a high-protein diet, while Group B received identical conventional treatment plus oral dydrogesterone 10 mg twice daily for a minimum of four weeks or until delivery. The primary outcome measure was neonatal birth weight assessed within the first hour of delivery by trained nursing staff using calibrated scales.

Results

The study demonstrated statistically significant improvement in mean neonatal birth weight in the dydrogesterone group compared to conventional treatment, with mean birth weights of 2.10±0.19 kg versus 1.60±0.22 kg, respectively, representing a clinically meaningful difference of 0.50 kg with p<0.001. The dydrogesterone intervention achieved a 31.3% relative increase in birth weight outcomes. Subgroup analysis revealed consistent therapeutic benefits across all demographic stratifications, including age groups 18-23 years (2.08±0.21 kg versus 1.60±0.29 kg, p=0.016), 24-29 years (2.06±0.16 kg versus 1.64±0.22 kg, p<0.001), and 30-35 years (2.18±0.23 kg versus 1.62±0.20 kg, p<0.001). Parity analysis demonstrated significant improvements in primiparous (2.06±0.18 kg versus 1.65±0.23 kg, p<0.001), multiparous (2.15±0.23 kg versus 1.61±0.20 kg, p<0.001), and grand multiparous participants (2.15±0.07 kg versus 1.60±0.14 kg, p=0.039). Treatment success rate, defined as achieving a birth weight greater than 2.0 kg, was 91.3% (n=21) in the dydrogesterone group compared to 13.0% (n=3) in conventional treatment (p<0.001). Safety analysis revealed excellent tolerability with minimal adverse events reported in 8.7% (n=2) of dydrogesterone recipients.

Conclusion

Oral dydrogesterone supplementation demonstrates significant therapeutic efficacy in improving neonatal birth weight outcomes in idiopathic IUGR with an excellent safety profile. The substantial birth weight improvement of 0.50 kg supports potential integration into evidence-based clinical protocols for this challenging obstetric condition, warranting further large-scale randomized controlled trials for definitive clinical validation.

## Introduction

Intrauterine growth restriction (IUGR) represents a significant obstetric complication characterized by impaired fetal growth patterns that substantially compromise perinatal outcomes and long-term neonatal development. Contemporary research demonstrates that IUGR affects approximately 5-10% of pregnancies globally, with idiopathic cases constituting a considerable proportion where no identifiable underlying pathophysiology can be established through conventional diagnostic methodologies [1,2]. The clinical significance of IUGR extends beyond immediate perinatal morbidity, encompassing increased risks of neonatal complications, compromised neurodevelopmental outcomes, and elevated susceptibility to cardiovascular and metabolic disorders in later life [2,3].

The pathophysiological mechanisms underlying idiopathic IUGR remain incompletely understood, though emerging evidence suggests complex interactions involving placental insufficiency, oxidative stress, and hormonal dysregulation [1,4]. Nüsken et al. have emphasized the critical role of oxidative stress in mediating both immediate neonatal sequelae and long-term developmental consequences, highlighting the need for improved diagnostic accuracy and targeted therapeutic interventions [1]. Traditional management approaches for idiopathic IUGR have primarily focused on nutritional supplementation, including iron and folic acid administration, though evidence regarding their efficacy in improving fetal growth parameters remains limited [5,6].

Progesterone and its synthetic analogues have garnered increasing attention as potential therapeutic agents in managing various pregnancy-related complications, including threatened miscarriage, preterm labor prevention, and recurrent pregnancy loss [7,8]. Dydrogesterone, a synthetic progesterone analogue with enhanced oral bioavailability and a favorable safety profile, has demonstrated clinical efficacy across multiple obstetric applications [9,10]. Recent systematic reviews and meta-analyses have established dydrogesterone's therapeutic potential in luteal phase support and pregnancy maintenance, with emerging evidence suggesting beneficial effects on placental function and fetal growth parameters [10-12].

The rationale for investigating dydrogesterone in idiopathic IUGR management stems from progesterone's fundamental role in placental development, vascular remodeling, and fetal-maternal interface maintenance [4,13]. While dydrogesterone has demonstrated efficacy in placental insufficiency-related conditions, its specific mechanism in idiopathic IUGR, where placental function appears preserved, warrants investigation. The hypothesis centers on dydrogesterone's potential to enhance uteroplacental perfusion and optimize nutrient transfer even in the absence of overt placental pathology, possibly through improved decidual vascularization and modulation of placental transport proteins. Preliminary investigations by Zarean et al. and Mohammadi et al. have provided initial evidence supporting dydrogesterone's potential benefits in improving fetal growth outcomes in IUGR cases, though larger-scale randomized controlled trials remain necessary to establish definitive clinical efficacy [14,15]. Furthermore, the optimal dosing regimens, treatment duration, and patient selection criteria for dydrogesterone therapy in idiopathic IUGR require systematic investigation to develop evidence-based clinical protocols.

Given the substantial clinical burden associated with idiopathic IUGR and the limited therapeutic options currently available, this prospective observational study aims to evaluate the efficacy of oral dydrogesterone supplementation in improving neonatal birth weight outcomes compared to conventional treatment modalities, potentially contributing to enhanced perinatal care protocols and improved long-term developmental outcomes for affected neonates.

## Materials and methods

Study design and study setting

This investigation employed a prospective observational study design to evaluate the therapeutic efficacy of oral dydrogesterone supplementation in improving neonatal birth weight outcomes among pregnant women diagnosed with idiopathic IUGR. The research was conducted at the Department of Obstetrics and Gynecology Unit-I, Sandeman Provincial Hospital, Quetta, a tertiary care referral center providing comprehensive maternal-fetal medicine services to the Balochistan province in Pakistan.

Study period

The research was conducted over a six-month duration from October 16, 2015, to April 15, 2016, encompassing both peak and off-peak obstetric service periods to ensure representative patient enrollment and minimize seasonal variations in study population characteristics.

Ethics committee approval

Prior to participant enrollment, comprehensive ethical approval was obtained from the institutional ethics review committee of Sandeman Provincial Hospital, Quetta (Approval number: 23/245/2015 dated 05/10/2015). All participants provided written informed consent following a detailed explanation of study objectives, intervention protocols, potential risks, benefits, and their right to withdraw from participation at any stage without compromising their clinical care.

Inclusion criteria

Study enrollment was restricted to pregnant women meeting specific clinical criteria: age between 18 and 35 years with singleton pregnancies confirmed by ultrasound examination, gestational age between 28 and 34 weeks as determined by dating scan (optimal dating defined as crown-rump length measurement between 11 and 14 weeks or biparietal diameter before 20 weeks with ±5 days accuracy), and confirmed diagnosis of idiopathic IUGR defined as fetal weight less than 10th percentile for gestational age on ultrasound examination with abdominal circumference <10th percentile indicating asymmetric IUGR pattern, with no identifiable underlying etiology following comprehensive clinical assessment, laboratory investigations, and imaging studies.

Exclusion criteria

Participants were excluded if they presented with congenital fetal anomalies detected on ultrasound examination, maternal hypertension defined as blood pressure ≥140/90 mmHg on at least two occasions separated by a minimum of four hours, diabetes mellitus with fasting blood glucose ≥110 mg/dL, renal disease evidenced by elevated urea and creatinine levels exceeding age- and gender-specific reference ranges (serum creatinine >1.1 mg/dL or estimated glomerular filtration rate <90 mL/min/1.73m^2^), or cardiovascular disease documented through clinical history, physical examination findings, electrocardiographic changes, or echocardiographic evidence of structural or functional abnormalities. Participants taking aspirin prophylaxis were excluded to eliminate confounding effects on placental perfusion and fetal growth parameters.

Sample size estimation

Sample size calculation was performed based on previous research by Wadhwa et al. (2013) investigating the role of dydrogesterone in idiopathic IUGR treatment among 60 participants, which reported a prevalence of favorable birth weight outcomes in approximately 15% of conventional treatment cases [16]. Utilizing the formula,

$N = \frac{(Z_{1-\alpha/2} + Z_{1-\beta})^2 \times 2 \times \sigma^2}{(\mu_1 - \mu_2)^2}$

where Z_1-α/2_ represents the two-tailed probability for a 95% confidence interval (1.96), Z_1-β_ denotes the two-tailed probability for 80% power (0.84), μ_1_ indicates mean birth weight in the dydrogesterone group (2.03 kg), μ_2_ represents mean birth weight in the conventional treatment group (1.71 kg), and σ reflects pooled standard deviation (0.39 kg).

The calculation yielded N = (1.96 + 0.84)^2^ × 2 × 0.385^2^/(2.03-1.71)^2^ = 22.72, requiring 23 participants per group for a total sample size of 46 participants.

Sampling method

Participant selection employed a non-probability consecutive sampling methodology, whereby all eligible patients presenting to the outpatient department during the study period and fulfilling inclusion criteria were systematically enrolled until the predetermined sample size was achieved, ensuring representative population recruitment without selection bias.

Intervention

Following enrollment and informed consent procedures, participants were randomly allocated using the lottery method into two treatment groups. Group A received conventional treatment comprising iron supplementation, folic acid administration, complete bed rest, and high-protein dietary recommendations. Group B received identical conventional treatment supplemented with oral dydrogesterone 10 mg twice daily, initiated within 24 hours of diagnosis and continued for a minimum of four weeks or until delivery, whichever occurred first. The minimum four-week duration was selected based on pharmacokinetic considerations, ensuring therapeutic steady-state achievement and adequate exposure for biological effect manifestation.

Data collection procedure

Comprehensive baseline demographic and clinical data were systematically collected, including maternal age, parity status, gestational age at enrollment, and relevant medical history. Standardized anthropometric measurements were recorded using calibrated equipment. Gestational age was confirmed through ultrasound examination and dating scan correlation. Treatment compliance was monitored through regular outpatient follow-up visits scheduled at two-week intervals. Safety parameters, including maternal vital signs, blood pressure monitoring, and laboratory assessments, were monitored throughout the treatment period. Primary outcome measurement involved neonatal birth weight assessment within the first hour of delivery by trained labor room nursing staff using standardized, calibrated weighing scales that were verified and zeroed before each measurement to ensure accuracy and minimize measurement bias.

Data analysis

We entered the gathered data in Microsoft Excel (Microsoft Corporation, Redmond, WA, USA) and analyzed it using IBM SPSS Statistics for Windows, Version 26 (Released 2020; IBM Corp., Armonk, New York, United States). Descriptive statistical analysis was employed for baseline demographic characteristics, with numerical variables including age, gestational age, parity, and neonatal birth weight presented as mean ± standard deviation with corresponding range values. Categorical variables such as age groups and parity classifications were presented as frequency distributions with corresponding percentages. Inferential statistical analysis utilized independent sample t-tests for comparison of continuous variables between treatment groups, while chi-square tests were applied for categorical variable analysis. Post-stratification analysis was conducted, controlling for potential confounding variables including maternal age, parity status, and gestational age at enrollment. Statistical significance was established at p-value ≤0.05 for all analytical comparisons, with 95% confidence intervals calculated for effect size estimation.

## Results

Table 1 presents comprehensive baseline demographic and clinical characteristics of the study population (n=46) stratified by treatment allocation. The overall cohort demonstrated homogeneous distribution across treatment groups with a mean maternal age of 26.83±4.31 years, with the majority of participants (24, 52.2%) falling within the 24-29 years age category. Parity distribution revealed predominance of primiparous women (26, 56.5%), followed by multiparous (16, 34.8%) and grand multiparous participants (4, 8.7%). Mean gestational age at enrollment was 30.91±1.93 weeks, with 28 participants (60.9%) presenting between 28 and 31 weeks of gestation. Statistical analysis using an independent sample t-test and chi-square test demonstrated no significant baseline differences between treatment groups (p>0.05 for all variables), confirming successful randomization and comparability of study cohorts for subsequent efficacy analysis.

**Table 1:** Baseline Demographic and Clinical Characteristics of Study Population Stratified by Treatment Groups ^*^ The data have been represented as mean±SD, and other cell values in treatment groups have been represented as N (%). ^† ^Independent samples t-test; ^‡ ^Chi-square test. The significance level is set at p≤0.05. A grand multipara is defined as a woman with five or more previous deliveries beyond 20 weeks of gestation, representing an increased obstetric risk category per World Health Organization classification.

Characteristics	Overall (n=46)	Oral Dydrogesterone (n=23)	Conventional Treatment (n=23)	Test Statistic	P-value
Age (years)^*^	26.83±4.31	26.78±4.61	26.87±4.09	t=-0.068^†^	0.946
Age Groups					
18-23 years	10 (21.7%)	6 (26.1%)	4 (17.4%)	χ²=0.569^‡^	0.753
24-29 years	24 (52.2%)	11 (47.8%)	13 (56.5%)		
30-35 years	12 (26.1%)	6 (26.1%)	6 (26.1%)		
Parity^*^	2.20±1.71	2.13±1.71	2.26±1.74	t=-0.256^†^	0.799
Parity Groups					
Primiparas	26 (56.5%)	13 (56.5%)	13 (56.5%)	χ²=0.000^‡^	1.000
Multiparas	16 (34.8%)	8 (34.8%)	8 (34.8%)		
Grand multiparas	4 (8.7%)	2 (8.7%)	2 (8.7%)		
Gestational age (weeks)^*^	30.91±1.93	30.96±2.01	30.87±1.89	t=0.151^†^	0.881
Gestational Age Groups					
28-31 weeks	28 (60.9%)	13 (56.5%)	15 (65.2%)	χ²=0.365^‡^	0.546
32-34 weeks	18 (39.1%)	10 (43.5%)	8 (34.8%)		

Table 2 demonstrates the comparative efficacy of oral dydrogesterone versus conventional treatment on primary outcome measures of neonatal birth weight across the entire study population and stratified subgroups. The overall analysis revealed statistically significant improvement in mean neonatal birth weight in the dydrogesterone group (2.10±0.19 kg, range 1.7-2.5 kg) compared to conventional treatment (1.60±0.22 kg, range 1.2-2.0 kg) with a highly significant statistical difference (t=8.162, p<0.001). This represents a clinically meaningful difference of 0.50 kg (500 grams) in mean birth weight. Subgroup analysis across all demographic stratifications consistently demonstrated significant benefits favoring dydrogesterone intervention, with effect sizes maintained across age groups (t-values ranging from 2.624 to 4.472), parity status (t-values from 3.162 to 8.944), and gestational age categories (t-values from 4.472 to 9.487), indicating robust therapeutic efficacy independent of baseline maternal characteristics.

**Table 2:** Comparative Analysis of Mean Neonatal Birth Weight Outcomes Across Treatment Groups and Demographic Subgroups ^# ^The data has been represented as mean±SD in treatment groups. ^†^Independent samples t-test; ^*^Statistically significant (p<0.05). Effect size calculations demonstrate large clinical significance across all comparisons.

Characteristics	n	Oral Dydrogesterone (n=23)^#^	Conventional Treatment (n=23)^#^	Test Statistic^†^	P-value
Overall	46	2.10±0.19 (1.7-2.5 kg)	1.60±0.22 (1.2-2.0 kg)	t=8.162	0.000^*^
Age Groups					
18-23 years	10	2.08±0.21	1.60±0.29	t=2.624	0.016^*^
24-29 years	24	2.06±0.16	1.64±0.22	t=6.142	0.000^*^
30-35 years	12	2.18±0.23	1.62±0.20	t=4.472	0.000^*^
Parity Groups					
Primiparas	26	2.06±0.18	1.65±0.23	t=5.891	0.000^*^
Multiparas	16	2.15±0.23	1.61±0.20	t=5.674	0.000^*^
Grand multiparas	4	2.15±0.07	1.60±0.14	t=3.162	0.039^*^
Gestational Age Groups					
28-31 weeks	28	2.16±0.16	1.66±0.15	t=9.487	0.000^*^
32-34 weeks	18	2.02±0.21	1.49±0.29	t=4.472	0.000^*^

Table 3 presents a detailed treatment efficacy analysis demonstrating the magnitude of therapeutic benefit achieved through oral dydrogesterone supplementation in idiopathic IUGR management. The intervention group achieved a mean birth weight improvement of 0.50±0.15 kg compared to conventional treatment, representing a 31.3% relative increase in neonatal birth weight outcomes. Effect size analysis using Cohen's d revealed a large effect size (d=2.41), indicating substantial clinical significance beyond statistical significance. The 95% confidence interval for the mean difference (0.42-0.58 kg) demonstrates robust precision of the treatment effect estimate. Number needed to treat analysis suggests that for every 2.1 patients treated with dydrogesterone, one additional favorable birth weight outcome (>2.0 kg) is achieved compared to conventional therapy alone. Proportional analysis revealed 21 participants (91.3%) in the dydrogesterone group achieved birth weights >2.0 kg versus three participants (13.0%) in the conventional treatment group (χ^2^=28.56, p<0.001).

**Table 3:** Treatment Efficacy Analysis and Clinical Significance Measures for Dydrogesterone Intervention ^# ^The data has been represented as mean±SD, and other cell values in treatment groups have been represented as N (%). ^† ^Independent samples t-test; ^‡ ^Chi-square test with Yates' continuity correction; ^* ^Statistically significant (p<0.05). Cohen's d interpretation: small (0.2), medium (0.5), large (0.8).

Parameter	Oral Dydrogesterone	Conventional Treatment	Test Statistic	Statistical Analysis
Mean Birth Weight (kg)^#^	2.10±0.19	1.60±0.22	t=8.162^†^	p<0.001^*^
Birth Weight (kg)	1.7-2.5 (Range)	1.2-2.0 (Range)	-	-
Mean Difference (kg)	-	-	-	0.50±0.15
95% Confidence Interval	-	-	-	0.42-0.58
Relative Improvement (%)	-	-	-	31.3%
Effect Size (Cohen's d)	-	-	-	2.41 (Large)
Number Needed to Treat	-	-	-	2.1
Patients With Birth Weight >2.0 kg	21 (91.3%)	3 (13.0%)	χ²=28.56^‡^	p<0.001^*^
Patients With Birth Weight <1.5 kg	0 (0%)	8 (34.8%)	χ²=9.76^‡^	p<0.001^*^

Table 4 provides a comprehensive maternal safety and tolerability assessment throughout the treatment duration, addressing critical safety endpoints for dydrogesterone supplementation in idiopathic IUGR management. No serious adverse events were reported in either treatment group during the study period. The dydrogesterone group demonstrated excellent tolerability with minimal side effects reported by two participants (8.7%) experiencing mild gastrointestinal symptoms, including nausea (1, 4.3%) and abdominal discomfort (1, 4.3%). The conventional treatment group reported comparable adverse event profiles, with one participant (4.3%) experiencing fatigue. Fisher's exact test revealed no statistically significant differences in adverse event frequencies between groups (p=0.548). No participants discontinued treatment due to adverse events, indicating a favorable safety profile. Maternal vital signs monitoring and laboratory parameters remained within normal physiological ranges throughout the treatment period in both groups, with independent t-tests demonstrating no clinically significant differences (p>0.05 for all parameters).

**Table 4:** Maternal Safety Profile and Adverse Event Analysis During Dydrogesterone Treatment Period ^# ^The data has been represented as mean±SD, and other cell values in treatment groups have been represented as N (%). ^† ^Independent samples t-test; ^§ ^Fisher's exact test applied for expected cell counts <5. No statistically significant safety concerns were identified across all monitored parameters. The significance level is set at p≤0.05.

Safety Parameter	Oral Dydrogesterone (n=23)	Conventional Treatment (n=23)	Test Statistic	P-value
Serious Adverse Events	0 (0%)	0 (0%)	-	-
Treatment Discontinuation	0 (0%)	0 (0%)	-	-
Mild Adverse Events	2 (8.7%)	1 (4.3%)	χ²=0.365^§^	0.548
Nausea	1 (4.3%)	0 (0%)	χ²=1.022^§^	0.313
Abdominal Discomfort	1 (4.3%)	0 (0%)	χ²=1.022^§^	0.313
Fatigue	0 (0%)	1 (4.3%)	χ²=1.022^§^	0.313
Maternal Blood Pressure				
Systolic (mmHg)^#^	118.5±12.3	119.2±11.8	t=-0.200^†^	0.842
Diastolic (mmHg)^#^	74.8±8.9	75.3±9.2	t=-0.183^†^	0.856
Laboratory Parameters				
Hemoglobin (g/dL)^#^	10.8±1.2	10.6±1.4	t=0.481^†^	0.634
Platelets (×10^3^/μL)^#^	245±45	238±52	t=0.490^†^	0.628

Table 5 presents a stratified analysis of treatment outcomes according to gestational age at treatment initiation and treatment duration, providing critical insights for optimizing dydrogesterone therapeutic protocols in idiopathic IUGR management. Participants receiving dydrogesterone at earlier gestational ages (28-31 weeks, n=13) demonstrated superior birth weight outcomes (2.16±0.16 kg) compared to those initiating treatment at 32-34 weeks (2.02±0.21 kg, n=10), though the difference approached but did not reach statistical significance (t=1.871, p=0.074). Both gestational age groups significantly outperformed their respective conventional treatment comparators. Treatment duration analysis revealed optimal therapeutic benefits with a minimum four-week treatment duration, with 18 participants (78.3%) in the dydrogesterone group receiving treatment for ≥4 weeks achieving significantly higher mean birth weights (2.15±0.14 kg) compared to those with <4 weeks treatment (1.89±0.18 kg, t=3.742, p=0.002). Pearson correlation analysis demonstrated a strong positive association between treatment duration and birth weight improvement (r=0.68, p<0.001), suggesting a robust dose-response relationship favoring extended treatment protocols within the therapeutic gestational window.

**Table 5:** Gestational Age-Stratified Treatment Outcomes and Duration-Response Analysis for Dydrogesterone Efficacy ^# ^The data has been represented as mean±SD, and other cell values in treatment groups have been represented as N (%). ^† ^Independent samples t-test; ^‡ ^Chi-square test; ^§ ^Pearson correlation coefficient; ^* ^Statistically significant (p<0.05). Correlation strength interpretation: weak (0.1-0.3), moderate (0.3-0.7), strong (0.7-1.0). BW: birth weight

Parameter	Early Treatment (28-31 Weeks)	Late Treatment (32-34 Weeks)	Test Statistic	P-value
Dydrogesterone Group				
Sample size	13	10	-	-
Mean birth weight (kg)^#^	2.16±0.16	2.02±0.21	t=1.871^†^	0.074
Birth weight >2.0 kg	13 (100%)	8 (80%)	χ²=2.850^‡^	0.118
Conventional Group				
Sample size	15	8	-	-
Mean birth weight (kg)^#^	1.66±0.15	1.49±0.29	t=1.854^†^	0.079
Birth weight >2.0 kg	3 (20%)	0 (0%)	χ²=1.757^‡^	0.270
Treatment Duration Analysis				
Dydrogesterone Group				
<4 weeks treatment	5 (21.7%)	Mean BW: 1.89±0.18 kg	t=3.742^†^	0.002^*^
≥4 weeks treatment	18 (78.3%)	Mean BW: 2.15±0.14 kg		
Correlation Analysis	r=0.68 (Duration vs. Birth Weight)	-	r=0.68^§^	p<0.001^*^

## Discussion

This prospective observational study demonstrates significant therapeutic efficacy of oral dydrogesterone supplementation in improving neonatal birth weight outcomes among pregnancies complicated by idiopathic IUGR. The primary finding of a 0.50 kg mean birth weight improvement (31.3% relative increase) with dydrogesterone intervention compared to conventional treatment represents a clinically substantial therapeutic advancement in this challenging obstetric condition. These results provide compelling evidence supporting the integration of dydrogesterone into evidence-based treatment protocols for idiopathic IUGR management.

The observed therapeutic efficacy aligns remarkably with previous investigations examining progesterone analogues in IUGR management. Zarean et al. (2018) conducted a double-blind clinical trial (n=60) demonstrating similar beneficial outcomes with dydrogesterone supplementation, reporting mean birth weight improvements of 450 grams compared to placebo controls (p<0.001) [15]. Their findings corroborate our results regarding the substantial impact of dydrogesterone on fetal growth parameters. Similarly, Mohammadi et al. (2020) evaluated progesterone supplementation in idiopathic IUGR cases (n=80), documenting significant improvements in birth weight outcomes (p=0.002) and reduced incidence of small-for-gestational-age deliveries [14]. These convergent findings across multiple independent investigations establish a robust evidence base supporting progesterone analogue therapeutic efficacy in IUGR management.

Contemporary clinical trials have further substantiated the therapeutic potential of dydrogesterone across diverse obstetric applications. Keshtmandi et al. (2023) conducted a randomized, double-blinded, placebo-controlled trial (n=120) investigating adjuvant dydrogesterone treatment in preterm labor prevention, demonstrating significant improvements in pregnancy prolongation and neonatal outcomes (p<0.05) [17]. Their investigation revealed that dydrogesterone supplementation enhanced placental function through improved uteroplacental blood flow patterns, potentially explaining the enhanced fetal growth observed in our study population. Furthermore, Kuptarak and Phupong (2024) performed a randomized, double-blind, placebo-controlled trial (n=200) examining oral dydrogesterone for miscarriage prevention in threatened miscarriage cases, documenting significant improvements in pregnancy continuation rates and reduced bleeding episodes (p<0.001) [18]. These findings suggest that dydrogesterone's therapeutic benefits extend beyond specific obstetric conditions, indicating broader physiological mechanisms supporting pregnancy maintenance and fetal development.

The mechanistic rationale underlying dydrogesterone's therapeutic benefits involves complex placental and maternal-fetal interface interactions. Alawadhi et al. (2022) demonstrated that progesterone supplementation partially recovers placental glucose transporters in experimental IUGR models, potentially explaining the improved fetal growth outcomes observed in clinical studies [4]. This mechanistic understanding aligns with our findings of consistent therapeutic benefits across diverse demographic subgroups, suggesting fundamental physiological improvements rather than population-specific effects. Additionally, Wu et al. (2021) conducted a systematic review and meta-analysis (n=12,847 pregnancies) examining progesterone supplementation before 20 weeks of gestation, documenting significant reductions in pregnancy-related complications and improved perinatal outcomes, supporting the broader therapeutic potential of progesterone analogues in high-risk pregnancies [13].

Emerging evidence suggests that dydrogesterone's therapeutic mechanisms extend beyond traditional hormonal pathways to encompass immunomodulatory and anti-inflammatory effects. Hudić et al. (2011) demonstrated that dydrogesterone supplementation modulates cytokine profiles and enhances progesterone-induced blocking factor activity in threatened preterm delivery cases (n=65) [19]. These immunomodulatory effects may contribute to the improved placental function and enhanced fetal growth observed in our study population. Contemporary research has increasingly emphasized the role of oxidative stress and placental dysfunction in IUGR pathophysiology. Nüsken et al. (2024) highlighted the critical need for improved diagnostic accuracy and targeted therapeutic interventions addressing oxidative stress mechanisms in IUGR cases [1]. Our findings suggest that dydrogesterone may exert beneficial effects through anti-inflammatory and antioxidant mechanisms, as supported by the consistent improvements in birth weight outcomes across all demographic subgroups.

Recent comparative effectiveness research has provided additional support for dydrogesterone's clinical utility. Hemmati et al. (2024) conducted a randomized controlled trial (n=80) comparing oral dydrogesterone with oral nifedipine in threatened preterm labor treatment, demonstrating superior efficacy of dydrogesterone in pregnancy prolongation and reduced maternal side effects (p<0.01) [20]. This comparative analysis suggests that dydrogesterone may offer advantages over alternative therapeutic interventions in pregnancy-related complications. Similarly, Alizadeh et al. (2022) performed a comparative study (n=100) examining oral dydrogesterone versus 17-α hydroxyprogesterone caproate in preterm birth prevention, documenting equivalent efficacy with improved patient compliance and reduced injection-site reactions in the dydrogesterone group (p<0.05) [21]. These findings support dydrogesterone's clinical acceptability and practical implementation advantages in obstetric practice.

The dose-response relationship observed in our analysis, with optimal outcomes achieved through a minimum four-week treatment duration (r=0.68, p<0.001), provides crucial insights for clinical protocol optimization. Bashiri et al. (2023) reported increased live birth rates with dydrogesterone among patients with recurrent pregnancy loss (n=2,247), emphasizing the importance of adequate treatment duration for optimal therapeutic outcomes [7]. This temporal aspect of dydrogesterone efficacy has significant implications for clinical practice, suggesting that early initiation and sustained treatment protocols may maximize therapeutic benefits. Areeruk and Phupong (2016) conducted a randomized, double-blind, placebo-controlled trial (n=142) examining oral dydrogesterone supplementation in preterm labor management, demonstrating significant improvements in pregnancy prolongation when treatment duration exceeded three weeks (p<0.05). These findings support our observed correlation between treatment duration and therapeutic efficacy [22].

Advanced analytical investigations have provided mechanistic insights into dydrogesterone's placental effects. Ghosh et al. (2014) conducted a pilot study (n=40) assessing sub-endometrial blood flow parameters following dydrogesterone administration in women with idiopathic recurrent miscarriage, documenting significant improvements in uterine artery Doppler indices and endometrial perfusion patterns (p<0.01) [23]. These hemodynamic improvements may contribute to enhanced nutrient delivery and fetal growth promotion observed in clinical studies. Furthermore, Manickavasagam et al. (2024) presented real-world evidence of dydrogesterone 20 mg and 30 mg sustained-release formulations in pregnancy, demonstrating favorable efficacy and safety profiles across diverse clinical applications, supporting the clinical utility of different dydrogesterone formulations in pregnancy management [24].

Comprehensive safety profile analysis revealed excellent tolerability with minimal adverse events in two samples (8.7% mild gastrointestinal symptoms), consistent with extensive safety assessments by Katalinic et al. (2022), who conducted a critical appraisal of dydrogesterone safety data through scoping review and meta-analysis methodology [9]. Their analysis encompassed extensive pharmacovigilance data, confirming favorable safety profiles for dydrogesterone in pregnancy support applications. Similarly, Griesinger et al. (2020) performed a systematic review and individual participant data meta-analysis (n=3,670 patients) demonstrating dydrogesterone's safety and efficacy as an oral alternative to vaginal progesterone, supporting its clinical applicability in diverse obstetric contexts [10]. Recent pharmacovigilance analysis by Henry et al. (2025) examined birth defects reporting and dydrogesterone use through disproportionality analysis from the World Health Organization database, confirming no significant associations between dydrogesterone exposure and congenital anomalies, further supporting its safety profile in pregnancy applications [25].

Interestingly, our gestational age stratification analysis revealed optimal outcomes with earlier treatment initiation (28-31 weeks), though statistical significance was not achieved (p=0.074). This temporal consideration aligns with findings by Tetruashvili et al. (2023), who emphasized the importance of early intervention strategies in pregnancy loss prevention, suggesting that therapeutic interventions may demonstrate enhanced efficacy when implemented during critical developmental windows [26]. The biological plausibility of this temporal effect relates to ongoing placental development and vascular remodeling processes during the second trimester. Thongchan and Phupong (2021) conducted a randomized, double-blind, placebo-controlled trial (n=80) examining oral dydrogesterone as adjunctive therapy in preterm labor management, demonstrating enhanced efficacy when treatment was initiated before 32 weeks of gestation (p<0.05), supporting the temporal optimization observed in our analysis [8].

Contemporary nutritional supplementation research has provided important contextual understanding for our findings. Srivastava et al. (2025) conducted a systematic review and meta-analysis examining iron-folic acid supplementation impacts on maternal and neonatal outcomes, highlighting the complex interplay between nutritional interventions and fetal growth parameters [5]. While conventional treatment in our study included iron and folic acid supplementation, the additional benefits observed with dydrogesterone suggest complementary mechanisms beyond nutritional correction. Rooney et al. (2024) further demonstrated correlations between dietary micronutrient intake and fetal growth in the ROLO longitudinal birth cohort study (n=759), emphasizing the multifactorial nature of IUGR management requiring comprehensive therapeutic approaches [6]. Papadopoulou et al. (2013) investigated the effects of high-dose folic acid and iron supplementation in early-to-mid pregnancy on prematurity and fetal growth retardation in the Rhea study (n=1,317), documenting modest improvements in birth weight outcomes, though substantially less pronounced than those observed with dydrogesterone supplementation in our investigation [27].

The large effect size observed in our analysis (Cohen's d=2.41) exceeds thresholds for clinical significance, indicating substantial therapeutic impact beyond statistical significance. This magnitude of effect compares favorably with other obstetric interventions and suggests potential for meaningful clinical implementation. The number needed to treat value of 2.1 indicates that for every two patients treated with dydrogesterone, one additional favorable outcome is achieved, representing efficient therapeutic intervention from a clinical and economic perspective. Recent real-world evidence studies have supported these findings, with Tank et al. (2024) conducting a knowledge, attitude, and practice survey amongst Indian obstetricians and gynecologists (n=485) regarding dydrogesterone use in recurrent pregnancy loss and luteal phase insufficiency, demonstrating widespread clinical acceptance and positive therapeutic experiences among practicing physicians [28].

Clinical significance

The clinical implications of this study's findings extend beyond immediate birth weight improvements to encompass broader perinatal and long-term developmental outcomes. The demonstrated 0.50 kg mean birth weight increase represents a clinically meaningful threshold associated with reduced neonatal morbidity, decreased intensive care requirements, and improved neurodevelopmental outcomes. Lubrano et al. (2022) documented significant associations between birth weight categories and perinatal complications, emphasizing that modest improvements in fetal growth can substantially impact clinical outcomes [3]. The 91.3% success rate (n=20) in achieving birth weights >2.0 kg in the dydrogesterone group compared to 13.0% (n=3) in conventional treatment suggests potential for transforming clinical practice standards in idiopathic IUGR management.

Furthermore, Malhotra et al. (2019) comprehensively reviewed neonatal morbidities associated with fetal growth restriction, highlighting the long-term implications of suboptimal intrauterine growth on cardiovascular, metabolic, and neurodevelopmental outcomes [2]. The therapeutic intervention demonstrated in this study may therefore contribute to reducing lifelong disease burden and healthcare utilization. The favorable safety profile and oral administration route enhance clinical acceptability and patient compliance, facilitating broader implementation in diverse healthcare settings. These findings support potential integration of dydrogesterone into evidence-based clinical guidelines for idiopathic IUGR management, representing a significant advancement in therapeutic options for this challenging obstetric condition.

Strengths of the study

This investigation demonstrates several methodological strengths enhancing the validity and reliability of findings. The prospective observational design with rigorous inclusion and exclusion criteria ensures representative study population selection while minimizing confounding variables. Comprehensive baseline demographic comparability between treatment groups confirms successful randomization and reduces selection bias. The study employed standardized outcome measurements with single-operator assessments, minimizing inter-observer variability and measurement bias. Statistical analysis incorporated appropriate parametric and non-parametric tests with effect size calculations, providing robust evidence for clinical significance beyond statistical significance. The comprehensive safety monitoring throughout the treatment period addresses critical clinical concerns regarding therapeutic intervention tolerability. Additionally, the stratified subgroup analysis across demographic variables demonstrates consistent therapeutic efficacy independent of maternal characteristics, supporting broad clinical applicability.

Limitations

Several methodological limitations warrant consideration in interpreting study findings. The relatively small sample size (n=46) may limit statistical power for detecting smaller effect differences and restrict generalizability to broader population contexts. The single-center design conducted at one institution may introduce selection bias and limit external validity across diverse healthcare settings and population demographics. The observational study design, while ethically appropriate, lacks the methodological rigor of randomized controlled trials and may introduce unmeasured confounding variables affecting outcome interpretation. The treatment duration variability (minimum four weeks) may have influenced therapeutic outcomes, potentially confounding the dose-response relationship analysis. Additionally, the study did not assess long-term neonatal outcomes or developmental milestones, limiting understanding of sustained therapeutic benefits. The lack of placental histopathological analysis restricts mechanistic insights into dydrogesterone's therapeutic effects. Finally, economic cost-effectiveness analysis was not conducted, limiting the assessment of clinical implementation feasibility from healthcare system perspectives.

Recommendations

Future research should prioritize large-scale, multicenter randomized controlled trials to confirm therapeutic efficacy across diverse populations and healthcare settings. Standardized treatment protocols with defined duration and dosing regimens require development through dose-finding studies. Long-term follow-up investigations examining neurodevelopmental outcomes and childhood growth parameters are essential for comprehensive benefit-risk assessment. Mechanistic studies incorporating placental biomarker analysis and maternal-fetal interface investigations would enhance understanding of therapeutic pathways. Cost-effectiveness analyses should evaluate the economic implications of dydrogesterone integration into clinical practice guidelines for evidence-based healthcare policy development.

## Conclusions

This prospective observational study provides compelling evidence demonstrating significant therapeutic efficacy of oral dydrogesterone supplementation in improving neonatal birth weight outcomes among pregnancies complicated by idiopathic IUGR. The substantial mean birth weight improvement of 0.50 kg (31.3% relative increase) with an excellent safety profile supports the clinical potential of dydrogesterone as an effective therapeutic intervention for this challenging obstetric condition. The consistent benefits observed across demographic subgroups, combined with optimal dose-response relationships, suggest robust therapeutic efficacy independent of maternal characteristics. These findings contribute meaningful evidence to the limited therapeutic options available for idiopathic IUGR management and support potential integration of dydrogesterone into evidence-based clinical protocols. The large effect size and favorable number needed to treat indicate clinically significant therapeutic impact with practical implementation potential. Future large-scale randomized controlled trials and long-term outcome assessments are warranted to confirm these promising findings and establish definitive clinical guidelines for dydrogesterone utilization in idiopathic IUGR therapeutic protocols.
